# Assessing the Risk of Peripheral Neuropathy Among Male Tobacco Smokers With Type 2 Diabetes: A Matched Case-Control Study

**DOI:** 10.1177/21501319251368607

**Published:** 2025-10-28

**Authors:** Sana Bader, Sanah Hasan, Rakibul M. Islam, Ghisson Abdulrazak, Khadija Al Zarouni, Mariam Muayyad, Md. Nazmul Karim

**Affiliations:** 1 School of Public Health and Preventive Medicine, Monash University, Melbourne, VIC, Australia; 2 Department of Clinical Sciences, College of Pharmacy and Health Sciences, Ajman University, Ajman, UAE; 3 Center of Medical and Bio-allied Health Sciences, Ajman University, Ajman, UAE; 4 University Hospital Sharjah, UAE; 5 Al Qassimi Women’s and Children’s Hospital, Sharjah, UAE

**Keywords:** diabetes mellitus, peripheral neuropathy, tobacco smoking

## Abstract

**Background and Aims:**

The increasing prevalence of diabetic complications and tobacco consumption are alarming worldwide. The aim of this study was to assess the association between tobacco smoking and diabetic peripheral neuropathy (DPN) among male smokers with type 2 diabetes.

**Research Design and Methods:**

In a matched case-control study, participants were recruited from 2 major hospitals in Sharjah, the United Arab Emirates (UAE). Male patients aged ≥18 years and diagnosed with DPN who had type 2 diabetes mellitus for at least 1 year were recruited. Age-(±3 years)-matched controls with diabetes were recruited for each case. Sociodemographic characteristics and self-reported data on physical activity, and tobacco smoking were collected. Smoking status was assessed as current, past, or never. Medical and clinical data were collected from hospital records.

**Results:**

An equal 140 of each cases and controls were included in the analysis, with a mean (SD) age of 63.8 (10.6) and 63.7 (10.5), respectively. Cases were more likely to smoke and had a longer smoking duration than the controls. After adjusting for all potential confounders, the association between tobacco smoking and DPN was significant (past smokers [OR = 4.10; 95% CI = 1.84-9.17], and current smokers [OR = 2.48; 95% CI = 1.06-5.83]).

**Conclusions:**

This study found a significant association between tobacco consumption and DPN among males with type 2 diabetes in the UAE. Targeted smoking cessation programmes are urgently needed.

## Introduction

Diabetes mellitus is a global public health concern that places a great burden on individuals’ health and the healthcare system alike.^1^ The prevalence of diabetes complications is alarmingly high, which can be microvascular (eg, neuropathy, retinopathy, and nephropathy) and macrovascular (eg, coronary artery disease, peripheral artery disease, and cerebrovascular disease).^2^ In 2021, around 12.2% of the global mortality from all causes was diabetes-related.^3^ Over the past 15 years, global health spending on diabetes has increased by 316%, driven by the rising burden of diabetes and its complications.^3^ Specifically in Gulf Cooperation Council (GCC), around 1.9% to 5.7% of the healthcare spending was diabetes-related.^4^

Diabetic peripheral neuropathy (DPN) is the most common among all diabetes complications,^2^ affecting nearly 50% of individuals with diabetes at some point in their lifetime.^5^ DPN accounts for 75% to 90% of all neuropathy cases in individuals with diabetes^6^ and leads to the highest hospitalisation rate among all diabetes complications combined.^7,8^ DPN worsens the burden of diabetes substantially, contributing to reduced quality of life^9^ and increasing the risk of foot ulcers, infections, amputation, and disability.^2,10^ Individuals with painful DPN are known to be at higher risk of developing mental disorders, including depression, anxiety, and sleep disturbance.^11^ The nerve damage induced by DPN is usually irreversible, highlighting the necessity of risk factor prevention and early detection to mitigate its devastating consequences.^12^

Non-modifiable risk factors for DPN have been proposed to include age at diagnosis, duration of diabetes, microalbuminuria, and macroalbuminuria^13,14^ while modifiable risk factors include tobacco smoking, high body mass index (BMI), and elevated glycosylated haemoglobin (HbA1C) levels.^13,14^ Evidence from various populations over the past 2 decades has reported a link between tobacco smoking and DPN,^15^ this is of great magnitude given the substantial prevalence of diabetes^3^ and widespread use of tobacco globally.^16,17^

In the Middle East, there were 73 million cases of diabetes in 2021, projected to reach 136 million by 2045, and indicating an 87% increase in diabetes prevalence.^3^ The prevalence of DPN is also notably high in the Middle East,^18-22^ with a varying prevalence between 9% and 61%,^23^ and nearly 5.3% of all persons with diabetes found to have neuropathy in UAE.^3^

According to the World Health Organization (WHO)^16^ 2020 report, the prevalence of tobacco consumption in the Eastern Mediterranean region, known as the Middle East, was 18.3%, with the vast majority of smokers being adult males. The case is relevant in a country such as the United Arab Emirates (UAE), where the use of tobacco products is considerably high among males, with a prevalence of 36% for the current use of any tobacco products and 29% for tobacco cigarettes.^24,25^

Existing evidence within this demographic region has focussed only on DPN and revealed that smoking is associated with an increased risk of DPN among individuals with diabetes.^26-29^ These studies also did not primarily focus on smoking as a risk factor for DPN; smoking was studied among other possible risk factors, which could compromise the findings from these studies due to the inclusion of multiple exposures and outcomes. Currently, there is scarcity of research focussing on sex-specific differences in the tendency to smoke and how smoking is associated with DPN in males. To our knowledge, no study exists on this association, particularly among male individuals with diabetes in in the Middle East, meaning that the association remains unexplored in this population. Thus, this case-control study aimed to examine the association between tobacco smoking and DPN among male individuals with type 2 diabetes.

## Research Design and Methods

### Study Design, Setting, and Participants

This matched case-control study was conducted in 2 major tertiary teaching hospitals in Sharjah, UAE. The data were collected between November 2023 and June 2024. The hospitals were purposively selected because they mostly served Arabic-speaking individuals with diabetes complications. Ethical approval was obtained from relevant authorities. Verbal informed consent was obtained from all participants prior to their partaking in the study. Conduct and reporting of this study adhered to the STROBE checklist (see Supplemental Material 1).

Male participants aged ≥18 years with a minimum of 1-year diagnosis of diabetes were included in the study. Those with neurological comorbidities (eg, previous stroke, congenital or genetic conditions with potential neurological involvement), peripheral vascular disease, or diagnosed with type 1 diabetes were excluded. Cases were defined as male adults with DPN diagnosis who were receiving treatment for diabetes at the designated hospital; controls were male patients with no DPN diagnosis who were also receiving treatment for diabetes at the same hospital. The inclusion and exclusion criteria were identical for cases and controls, except for DPN diagnosis, which was only a feature for cases. Subsequently, 1 control per case was selected by matching age (±3 years) from the databases of the 2 hospitals. The diagnosing physicians used the following criteria to diagnose participants for DPN: Careful history and presence of temperature and/or pinprick sensation for small fiber function, while 128 Hz for vibration sensation for large fiber function, were used to assess distal symmetric polyneuropathy.

### Strategies to Mitigate Potential Biases

To reduce selection bias, cases and controls were systematically screened and recruited from the same source, the 2 major hospitals participating in the study, using predefined eligibility criteria. Controls were matched to the cases by age to ensure that all participants were from the same source. Moreover, information bias was minimised by using a validated questionnaire to assess smoking history (Global Tobacco Surveillance System), which was administered by trained research assistants who interviewed each participant following a standardised question. However, the research assistants were not blinded to the case and control groups and following a predefined category of smoking status (current, past, or never smoker) improved the consistency and reliability of the exposure evaluation.

### Study Instrument and Data Collection

The study instrument consisted of 2 parts: the first part captured self-reported data including participant sociodemographic characteristics, tobacco smoking history using the Global Tobacco Surveillance System, and physical activity using the Global Physical Activity Questionnaire (GPAQ). The second part consisted of clinical and biochemical data obtained from the participants’ electronic medical records at the hospital.

Self-reported sociodemographic characteristics included participant age, sex, education level, family history of diabetes mellitus, their own diabetes type, and years since diagnosis. Participants’ smoking habits using items adapted from the Global Tobacco Surveillance System by the WHO were also collected.^30^ Participants were asked to indicate their smoking status (current, past, or never smoker), frequency of smoking (daily or less), preferred method of tobacco consumption, duration of smoking, age at initiation of smoking, and any attempts at smoking cessation. The participants’ physical activity was assessed using a validated instrument by the WHO, the Global Physical Activity Questionnaire (GPAQ).^31^ Patients’ physical activity was classified as sufficient or insufficient in relation to their achieved scores on the GPAQ.

The second part of the instrument consisted of clinical and biochemical data obtained from patient’s medical records which confirmed a clinical diagnosis of diabetes and included participant information such as height, weight, diagnosis of DPN, hypertension, and dyslipidaemia, use of herbal supplements current and previous diabetes treatment including use of insulin, and the presence of diabetes complications. The study instrument was uploaded on secure web-based software, Qualtrics XM to collect the data. The questionnaire is provided in Supplemental Material 2.

Data collection took place in the outpatient clinics of the participating hospitals. Secure computer access was provided to trained research assistants on the project. A list of patients who were seen for type 2 diabetes mellitus and diabetic peripheral neuropathy was obtained. The research assistants accessed the patients’ medical records and further screened each patient’s medical profile against the study selection criteria. Those who were eligible were approached through their contact details (obtained from the medical records) and were called by telephone to gauge their interest to participate in the study. Those agreeing gave verbal consent and responded to questions asked by the research assistants. Data collected included demographics and self-reports smoking behavior and physical activity. Participant medical and clinical data were collected from the medical records available through secure computer access. Data collection continued until the required number of observations with complete information on all relevant variables was achieved. As a result, cases with missing data on key exposure, outcome, or covariate variables were excluded from the final analysis.

### Sample Size

Considering that this is a 1:1 matched case-control study design, a minimum sample size of an equal 140 of each cases and controls is required to achieve 80% statistical power to detect an odds ratio (OR) of 2 at a 95% confidence level. The sample size was calculated using the following formula for matched case-control studies:

$$n=\left(\frac{{\left(Z\;\alpha /2+Z\beta \right)}^{2}\times \left(p0\left(1-p0\right)+p1\left(1-p1\right)\right)}{{\left(p1-p0\right)}^{2}}\right)$$

Where:

- *Z*_α/2_ = 1.96 for a 2-sided significance level of 0.05
- *Z*_β_ = 0.84 for 80% power
- *p*_0_ is the expected prevalence of exposure among controls
- *p*_1_ is the expected prevalence of exposure among cases, derived from the assumed odds ratio (OR)

We assumed an exposure prevalence (*p*0) of 0.30 among controls based on findings from previous literature in similar populations. Given an OR of 2.0, the corresponding prevalence among cases (*p*1) was calculated using the following formula:

$$p1=\frac{\mathrm{OR}\times p0}{1+p0\times \left(OR-1\right)}=\frac{2\times 0.30}{1+0.30\times \left(2-1\right)}=0.46$$

Substituting these values into the sample size formula yielded a minimum of approximately 140 matched pairs (ie, 140 cases and 140 controls) to detect an OR of 2.0 with 80% power at a 95% confidence level. We selected an OR of 2.0 as the effect size of interest based on both clinical relevance and findings from previous studies, where this magnitude of association was commonly reported and considered meaningful in public health and clinical contexts.

### Statistical Analysis

Descriptive statistics were generated to compare patient characteristics between cases and controls. Differences were assessed using the chi-square test for categorical data and independent sample t-test for numerical data to identify potential confounders, including age, BMI, educational level, physical activity, presence of chronic comorbidities, early onset of DM, duration of DM, HbA1C%, insulin as initiation therapy, current insulin therapy, and presence of any diabetic complications. The association between smoking status and DPN was examined using multivariable conditional logistic regression, adjusted for plausible confounders. Adjusted odds ratios and 95% confidence intervals were reported. Our regression model was built using a conceptual and data-driven approach. Variables were selected as potential confounders based on the prior literature, theoretical relevance, and associations observed in the bivariate analysis.

To assess multicollinearity, we calculated uncentred Variance Inflation Factors (VIFs) for the variables included in the final 4 multivariable logistic regression models. A VIF value greater than 10 was considered indicative of multicollinearity. To enhance model stability and interpretability, we re-estimated the affected models after excluding the identified collinear variable(s).

All statistical tests were 2-sided, and *P* < .05 was considered statistically significant.

Data were analysed using the Statistical Software for Data Science (Stata Corporation, College Station, TX, USA), Version 17.

## Results

Of 316 male patients approached, 280 agreed to participate in this study and completed the telephone interviews (response rate = 88.6%); thus, data from 280 patients were analysed.

The mean (SD) age for the case was 63.8 (10.6) and for controls was 63.7 (10.5; Table 1).

**Table 1.** Characteristics of Study Participants (N = 280).

Characteristics	Cases	Controls	Total	*P*-value
Age in years – mean (Sd)	63.75 (10.61)	63.69 (10.50)	63.72 (10.54)	.96
Educational level				
High School – n (%)	95 (67.86)	84 (60)	179 (63.93)	.171
Graduate and above – n (%)	45 (32.14)	56 (40)	101 (36.07)	
BMI categories (kg/m^2^) – n (%)				
Normal (<25)	24 (17.14)	28 (20)	52 (18.57)	.182
Overweight (25-30)	55 (39.29)	66 (47.14)	121 (43.21)	
Obese (>30)	61 (43.57)	46 (32.86)	107 (38.21)	
Sedentary activity per/min/day – mean (SD)	936.46 (181.05)	951 (133.02)	944.02 (158.83)	.43
Physical activity – n (%)				
Sufficient (>600 MET)	23 (16.43)	30 (21.43)	53 (18.93)	.286
Insufficient (<600 MET)	117 (83.57)	110 (78.57)	227 (81.07)	
Dyslipidaemia – n (%)				
Yes	129 (92.14)	123 (87.86)	252 (90)	.232
No	11 (7.86)	17 (12.14)	28 (10)	
Presence of major comorbidities – n (%)				
Yes	108 (77.14)	94 (67.14)	202 (72.14)	.062
No	32 (22.86)	46 (32.86)	78 (27.86)	
Family history of DM – n (%)				
Yes	98 (70.00)	108 (77.14)	206 (73.57)	.175
No	42 (30)	32 (22.86)	74 (26.43)	
Early onset of DM – n (%)				
Yes	39 (28.06)	21 (15)	60 (21.51)	.008*
No	100 (71.94)	119 (85)	219 (78.49)	
Duration of DM – n (%)				
Newly diagnosed	12 (8.63)	25 (17.86)	37 (13.26)	.046*
Short duration	24 (17.27)	31 (22.14)	55 (19.71)	
Intermediate duration	42 (30.22)	39 (27.86)	81 (29.03)	
Long duration	61 (43.88)	45 (32.14)	106 (37.99)	
HbA1C% – n (%)				
<7%	64 (45.71)	60 (42.86)	124 (44.29)	.630
≥7%	76 (54.29)	80 (57.14)	156 (55.71)	
Insulin as initiation therapy – n (%)				
Yes	17 (12.14)	9 (6.43)	26 (9.29)	.100
No	123 (87.86)	131 (93.57)	254 (90.71)	
Current insulin therapy – n (%)				
Yes	52 (37.14)	26 (18.57)	78 (27.86)	.001*
No	88 (62.86)	114 (81.43)	202 (72.14)	
Presence of DM complications (microvascular or/and macrovascular) – n (%)				
Yes	95 (67.86)	59 (42.14)	154 (55)	<.001*
No	45 (32.14)	81 (57.86)	126 (45)	
Presence of diabetic nephropathy – n (%)				
Yes	50 (35.71)	26 (18.57)	76 (27.14)	.001*
No	90 (64.29)	114 (81.43)	204 (72.86)	
Presence of diabetic retinopathies – n (%)				
Yes	64 (45.71)	35 (25)	99 (35.36)	<.001*
No	76 (54.29)	105 (75)	181 (64.64	
Presence of diabetic foot/ ulcer – n (%)				
Yes	6 (4.29)	6 (4.29)	12 (4.29)	1.000
No	134 (95.71)	134 (95.71)	268 (95.71)	
Herbal supplements consumption – n (%)				
Yes	8 (5.71)	2 (1.43)	10 (3.57)	.053
No	132 (94.29)	138 (98.57)	270 (96.43)	
Vitamin B supplement intake – n (%)				
Yes	100 (71.43)	61 (43.57)	161 (57.50)	<.001*
No	40 (28.57)	79 (56.43)	119 (42.50)	

Sedentary activity per/min/day represent the total sedentary time per day in minutes.

*P < .05.

Participants in the case group had lower tertiary education attainment (32.14% vs 40.0%), longer diabetes duration (*P* = .046), and more frequently reported diabetes-related complications (*P* < .001) than those in the control group. The prevalence of obesity was also higher in the case group (43.57%) than in the control group (32.86%).

Diabetic microvascular complications were more frequently reported in the case group: retinopathy was present in nearly half of the cases (45.71%) and nephropathy in 35.71%. Similarly, the presence of chronic comorbidities (77.14% vs 67.14%) and dyslipidaemia (92.14 vs 87.86) was more frequent in cases than in controls. Compared to the controls, the cases reported a higher rate of insulin as initiation therapy (12.14% vs 6.43%) and as their current diabetes treatment modality (37.14% vs 18.57%).

Participants in the case group were more likely insufficiently activity (83.57%) while sedentary lifestyle were more commonly observed in the control group (average sitting hours per day = 15.85). Table 1.

Cases were significantly more likely to have smoked in comparison to controls (24.29% were current smokers and 36.43% were past smokers among cases, vs 13.57% current smokers and 20.71% past smokers among controls). Approximately 83.02% of current smokers smoked daily, 64.71% smoked tobacco cigarettes, 23.53% smoked waterpipes (also called shisha), and 11.76% smoked e-cigarettes.

Moreover, there was a statistically significant difference between the initial age of smoking (*P* = .0001) and “regular” smokers age (*P* = .0001) between the case and control groups (for both, past and current smoking). Overall, cases had a longer duration of smoking than the control (*P* ≤ .001; Table 2).

**Table 2.** History of Smoking.

Smoking history	Cases	Controls	Total	*P*-value
Current smokers – n (%)	34 (24.29)	19 (13.57)	53 (18.93)	<.001*
Past smokers – n (%)	51 (36.43)	29 (20.71)	80 (28.57)	
Smoking initiation age – mean (SD) – past and current smokers	12.95 (12.32)	7.44 (11.14)	10.19 (12.04)	.0001*
Regular smoking age – mean (SD) – past and current smokers	14.19 (13.70)	7.86 (12.18)	11.03 (13.32)	.0001*
Smoking cessation age – mean (SD) – past-smokers	49.22 (10.71)	46.55 (14)	48.25 (11.99)	.3426
Duration of smoking in years – mean (SD)- past and current smokers	18.06 (17.96)	9.65 (15.79)	13.85 (17.40)	<.001*
Frequency of smoking in current smokers – n (%)				
Less than daily	6 (17.65)	3 (15.79)	9 (16.98)	.863
Daily	28 (82.35)	16 (84.21)	44 (83.02)	
Type of tobacco smoking consumed among current smokers – n (%)				
Cigarette	22 (64.71)	13 (68.42)	35 (66.04)	.737
Shisha	8 (23.53)	5 (26.32)	13 (24.53)	
E-cigarette	4 (11.76)	1 (5.26)	5 (9.43)	

Smoking initiation age was defined as the age at which the participants first smoked ever and was determined based on the question, how old were you when you first smoked ever?

Regular smoking age was defined as the age at which the participant became a regular smoker and was determined based on the question, how old were you when you started smoking regularly?

*P < .05.

Table 3 presents the results of univariate and multivariate Logistic regression analyses of the association between smoking and DPN. Factors that showed significant associations with DPN in the univariate logistic regression included past (OR = 3.31; 95% CI = 1.75-6.27) and current smoking (OR = 3.31; 95% CI = 1.62-6.78), presence of chronic comorbidities (OR = 1.88; 95% CI = 1.02-3.44), early onset of diabetes (OR = 2.8; 95% CI = 1.36-5.76), longer duration of diabetes (OR = 3.17; 95% CI = 1.32-7.62), current insulin use (OR = 2.63; 95% CI = 1.48-4.67), presence of diabetes complications (OR = 2.89; 95% CI = 1.72-4.88), retinopathy (OR = 2.45; 95% CI = 1.46-4.12), and nephropathy (OR = 2.6; 95% CI = 1.43-4.72).

**Table 3.** Association of DPN With Smoking Adjusted for Plausible Predictors in Conditional Logistic Regression Analyses.

	Univariate	Model 1	Model 2	Model 3	Model 4
Predictors	OR (95% CI)	OR (95% CI)	OR (95% CI)	OR (95% CI)	OR (95% CI)
History of smoking^a^					
Past smoker	3.31 (1.75-6.27) **	3.47 (1.74-6.93)**	3.64 (1.80-7.37)**	3.53 (1.70-7.36)**	4.10 (1.84-9.17)**
Current smoker	3.31 (1.62-6.78) **	3.25 (1.56-6.77)**	2.91 (1.37-6.18)**	2.69 (1.19-6.05)*	2.48 (1.06-5.83)*
BMI categories (kg/m^2^)^b^					
Overweight (25-30)	1 (0.51-1.98)	0.70 (0.32-1.55)	0.64 (0.29-1.44)	0.70 (0.30-1.62)	0.47 (0.19-1.18)
Obese (>30)	1.66 (0.81-3.41)	1.20 (0.53-2.75)	1.18 (0.51-2.74)	1.27 (0.53-3.08)	0.95 (0.38-2.37)
Educational level^c^					
Graduate and above	0.68 (0.40-1.15)	0.72 (0.41-1.27)	0.71 (0.39-1.27)	0.59 (0.31-1.14)	0.59 (0.30-1.16)
Physical activity^d^					
Insufficient PA	1.37 (0.76-2.47)		0.93 (0.47-1.81)	0.74 (0.36-1.53)	0.72 (0.33-1.59)
Have a comorbidity diagnosis^e^	1.88 (1.02-3.44) *		1.95 (0.97-3.89)	1.87 (0.90-3.88)	1.57 (0.73-3.37)
Early onset DM^f^	2.8 (1.36-5.76) **			2.15 (0.84-5.52)	1.63 (0.58-4.60)
DM duration^g^					
Short duration	1.57 (0.62-3.97)			1.96 (0.65-5.87)	1.90 (0.57-6.32)
Intermediate duration	2.17 (0.93-5.09)			2.32 (0.84-6.43)	1.80 (0.60-5.43)
Long duration	3.17 (1.32-7.62) **			2.69 (0.88-8.22)	1.40 (0.41-4.78)
HbA1C (≥7%)^h^	0.89 (0.55-1.43)			0.81 (0.45-1.45)	0.66 (0.35-1.24)
Insulin as initiation therapy^i^	1.89 (0.84-4.24)				1.66 (0.55-5.03)
Current insulin therapy^j^	2.63 (1.48-4.67) **				1.62 (0.72-3.66)
Have any DM complication^k^	2.89 (1.72-4.88) **				2.52 (1.29-4.94) **
Diabetic retinopathy diagnosis^l^	2.45 (1.46-4.12) **				
Diabetic nephropathy diagnosis^m^	2.6 (1.43-4.72)**				

Model 1 adjusted for BMI categories, educational level.

Model 2 adjusted for BMI categories, educational level, physical activity, presence of any major comorbidity.

Model 3 adjusted for BMI categories, educational level, physical activity, presence of any major comorbidity, early onset of DM, Duration of DM, HbA1C% categories.

Model 4 adjusted for BMI categories, educational level, physical activity, presence of any major comorbidity, early onset of DM, Duration of DM, HbA1C% categories, starting insulin, current insulin, any diabetic complications.

Dependent variable – DPN reference categories ^a^Never smoker, ^b^BMI (normal), ^c^High school, ^d^Sufficient PA, ^e^No comorbidity, ^f^No early onset of DM, ^g^Newly diagnosed, ^h^HbA1C <7%, ^i^No Insulin initiation at diabetes diagnosis, ^j^No current use of insulin, ^k^No diagnosis of DM complications, ^l^No diagnosis of retinopathy, ^m^No diagnosis of nephropathy.

*P < .05. **P < .01.

After adjusting for all plausible predictors (BMI, educational level, physical activity, presence of chronic comorbidities, early onset of DM, duration of DM, HbA1C%, Insulin as initiation therapy, current insulin therapy, and presence of any diabetic complications), a higher risk of DPN was reported among past and current smokers ([OR = 4.10; 95% CI = 1.84-9.17] and [OR = 2.48; 95% CI = 1.06-5.83], respectively). The presence of diabetic complications was also significantly associated with DPN (OR = 2.52; 95% CI = 1.29-4.94).

## Discussion

This study demonstrated an association between tobacco smoking and DPN in male participants with type 2 diabetes. Despite that some literature had reported on this association,^26-29^ these previous studies did not solely explore the relationship between tobacco smoking and DPN, but rather reported smoking among the other risk factors for DPN,^26-28^ or other microvascular/macrovascular complications^29^; furthermore, none have reported on sex-specific assessments of smoking and DPN.^26-29^

This study provided new insights into the relationship between tobacco smoking and diabetic peripheral neuropathy (DPN) among male smokers in the Middle East. It highlighted that smoking significantly increases the risk of developing DPN in this population, a connection that has been previously underexplored. These findings underscore the importance of targeted interventions to reduce smoking rates among male patients with diabetes to mitigate the risk of DPN and improve their overall health outcomes.

Tobacco smoking may lead to DPN by inducing oxidative stress, which is believed to be the primary cause of cellular damage in diabetic neuropathy.^32^ Tobacco smoking may also lead to diabetic neuropathy by inducing insulin resistance.^15,33^ Insulin resistance has been shown to be independently associated with DPN and other neuropathy types in individuals with type 2 diabetes mellitus,^34^ regardless of glycaemic control. Tobacco smoking may also impair glycaemic control, which is believed to be the foundational approach for preventing or delaying DPN.^35^ Even though it is thought that glycaemic control is the ideal approach to prevent microvascular complications in individuals with diabetes mellitus,^36,37^ this does not seem sufficient to prevent DPN in the presence of tobacco smoking^15^; even after adjusting for HbA1C levels in our analysis, smoking remained a major risk factor for DPN, and glycaemic control was not associated with less DPN in the presence of smoking.

Our results showed that participants in the case group had a longer smoking duration than those in the control group (*P* < .001). Supporting evidence from a 6-year follow-up study which revealed an independent risk of long smoking duration and DPN.^38^ Additionally, this study found that both current and past smoking were risk factors for DPN. However, a higher risk was observed among past smokers, similar to recent findings of a meta-analysis that demonstrated past smoking as an independent risk factor for DPN.^39^ This result might also reflect that smoking-induced nerve damage is less likely to be reversible, attributable to the length of smoking, intensity, and duration since quitting smoking.^15^ Therefore, early smoking cessation intervention is due here to prevent long term smoking exposure which could lead to this irreversible nerve damage.

The influence of smoking cessation on diabetic complications has recently been reported in the literature, and smoking cessation was found to be associated with a reduced risk of diabetes-related complications.^40^ Previous studies have emphasised the need to develop and implement smoking cessation programmes tailored to individuals with diabetes.^41,42^ However, a personalised smoking cessation programme, which is condition- and sex-specific, has yet to be established. Despite current global evidence, a targeted cessation programme for males with diabetes and related complications remains unestablished. This calls attention to the urgent need to implement and provide supportive cessation services tailored to these individuals’ condition, needs and preferences, and the need for health professionals to engage in the process. Moreover, understanding facilitators of smoking and what is perceived as a barrier to quitting smoking in this population is essential as these may differ from those in the general population.^43^

Addressing sex-specific differences should also be considered when designing personalised cessation programmes, as each gender has its own motivation to smoke, cease smoking, or seek cessation services.^44^ It was thought that compared to females, males are more sensitive to the reinforcing effects of nicotine, obtain higher benefit from nicotine replacement therapy (NRT) while trying to quit, and are not as sensitive to the non-nicotinic effects of cigarettes.^45^ A supporting review of global evidence revealed that males who received NRTs reported a higher quit rate than females as males were found to have higher nicotine dependence scores; hence, they were more likely to smoke due to nicotine addiction.^46^ Another factor in quitting smoking among males is that smoking is perceived as an identity of masculinity and resilience among men, therefore, designing masculine-sensitive method of quitting smoking may be helpful.^46^ Among Arab males specifically,^47^ the main barriers that hinder smoking cessation were mainly related to external influences, such as the urge to smoke during social gatherings, the high accessibility of smoking products in public, stress, and the lack of time to join smoking cessation programmes,^47,48^ emphasising the effect of culture and social surrounding on smoking cessation, and the need to address these barriers in any programme designed for these patients. Considering the rising prevalence of smoking among females in the UAE,^24^ which is likely to result in a possible increase in the burden of DPN among females, it is crucial to address the issue of smoking-related stigma encountered by female smokers and the consequent impact on their behavioural health and well-being when designing a personalised smoking cessation intervention. A systematic review of the global literature on smoking-related stigma among females showed that females are more likely to be stigmatised by their family, healthcare providers, and social media which, in turn, might lead to social distress and hinder their motivation to cease smoking or seek cessation services.^49^ A similar finding from Al Kuwait, a country in the Middle East, suggested that smoking in males was foreseen as the norm, whereas it was not acceptable for females to smoke from a cultural perspective, forcing females to not smoke or admit smoking in public areas.^50^ Personalised smoking cessation should be promoted at any level of patient encounter within the healthcare system, and it does not only need to be in the clinic. In a recent study that evaluated the quality of smoking cessation counselling in community pharmacies in the UAE, Al Zubaidi et al^51^ proposed community pharmacies as a potential venue which offered patient-centred counselling to facilitate the dissemination of, and accessibility to smoking cessation.

To the best of our knowledge, this is the first case-control study conducted in the Middle East to assess the association between tobacco smoking and peripheral neuropathy among male smokers with diabetes. The major strength of the study was the outcome assessment; DPN cases were diagnosed by a physician, and hospital medical records were used to retrieve medical diagnoses and clinical data. Another strength of this paper was the assessment of lifetime smoking exposure meant to minimise bias that may arise from length of exposure. Smokers with diabetes may quit smoking after the development of peripheral neuropathy which could lead to underestimation of the association. One limitation of this study was the use of self-reported exposure which could have been contaminated by forgetfulness and/or social desirability; however, the use of validated tools developed by the WHO was likely to minimise the impact. Despite that the cases were matched to controls by the key confounder (age) and adjusting for other confounders in the analysis was important to consider, the effect of residual confounders could not be ruled out. Despite the potential biases in our study, these limitations do not undermine the importance of the findings and the knowledge gained from this study. Our study provided insights that can significantly enhance diabetes management in patients with type 2 diabetes. Furthermore, this study recruited participants from 2 major tertiary teaching hospitals’ outpatient clinics in the UAE. The characteristics of our study participants resembled those of smokers with T2DM, indicating that our findings might be generalisable to the broader community of male individuals with T2DM in the UAE.

## Conclusion

This study found a significant increased risk of DPN among male tobacco smokers with T2DM in the UAE. Future research should target a more in-depth assessment of the risk of peripheral neuropathy among smokers with diabetes, posed by the types and quantities of smoking. Additionally, Smokers with diabetes may greatly benefit from personalised smoking cessation programmes through integration into diabetes management care in healthcare systems.

## Supplemental Material

sj-pdf-1-jpc-10.1177_21501319251368607 – Supplemental material for Assessing the Risk of Peripheral Neuropathy Among Male Tobacco Smokers With Type 2 Diabetes: A Matched Case-Control Study Supplemental material, sj-pdf-1-jpc-10.1177_21501319251368607 for Assessing the Risk of Peripheral Neuropathy Among Male Tobacco Smokers With Type 2 Diabetes: A Matched Case-Control Study by Sana Bader, Sanah Hasan, Rakibul M. Islam, Ghisson Abdulrazak, Khadija Al Zarouni, Mariam Muayyad and Md. Nazmul Karim in Journal of Primary Care & Community Health

sj-pdf-2-jpc-10.1177_21501319251368607 – Supplemental material for Assessing the Risk of Peripheral Neuropathy Among Male Tobacco Smokers With Type 2 Diabetes: A Matched Case-Control Study Supplemental material, sj-pdf-2-jpc-10.1177_21501319251368607 for Assessing the Risk of Peripheral Neuropathy Among Male Tobacco Smokers With Type 2 Diabetes: A Matched Case-Control Study by Sana Bader, Sanah Hasan, Rakibul M. Islam, Ghisson Abdulrazak, Khadija Al Zarouni, Mariam Muayyad and Md. Nazmul Karim in Journal of Primary Care & Community Health
