# Supplementary material for: Assessing the Risk of Peripheral Neuropathy Among Male Tobacco Smokers With Type 2 Diabetes: A Matched Case-Control Study
Source: J Prim Care Community Health. 2025 Oct 28;16:21501319251368607. doi: 10.1177/21501319251368607 (PMC12576162; doi:10.1177/21501319251368607)
Supplement: sj-pdf-2-jpc-10.1177_21501319251368607 – Supplemental material for Assessing the Risk of Peripheral Neuropathy Among Male Tobacco Smokers With Type 2 Diabetes: A Matched Case-Control Study [file sj-pdf-2-jpc-10.1177_21501319251368607.pdf]

Supplementary Material 2: Questionnaire

Dear participants, thank you for agreeing to participate in this research study. You are invited to complete a survey to assess the effect of tobacco smoking on diabetic peripheral neuropathy. The survey is five pages long and should take you no more than 30 minutes to complete. We appreciate your thoughtful response in this survey.

Before you start the survey, kindly provide us with your preferred contact number: _______

SECTION A: Sociodemographic characteristics data

1. **What is your current age?** ____________ years

2. **What is your gender?** ____________

3. **Ethnicity:**
   0 Arab 1 Non-Arab

4. **What is your education level? (Year of formal schooling)**
   1. *Tertiary school or less*: any level of education less than undergraduate degree.
   2. *Undergraduate:* successfully completed an undergraduate degree level course at a college or university.
   3. *Higher degree:* a degree that is higher than bachelor's degree.

5. **Family history of diabetes?**
   0 No first-degree relatives with family history of diabetes
   1 First-degree relatives with a family history of diabetes

6. **Which type of diabetes are you diagnosed with?**
   1. Type 1 diabetes mellitus
   2. Type 2 diabetes mellitus

7. **What was your age at diabetes diagnosis?** _____________ Years

8. **How long have you been diagnosed with diabetes?** _______________ years

9. **Are you diagnosed with diabetic <u>neuropathy by a physician</u>?**
   0 No 1 Yes. If yes specify type _____________ and age at diagnosis ______ years.

10. **Are you diagnosed with high blood pressure?**
    0 No 1 Yes

11. Do you have any other chronic conditions (diagnosed and being treated)?

1. Heart diseases – if yes specify name ___________________
2. Kidney diseases – if yes specify name __________________
3. Cancers – if yes specify name __________________
4. Respiratory disease – if yes specify name __________________
5. Other disease – if yes specify name _____________
6. None

.

SECTION B: Clinical and biochemical variables.

12. The following data will be obtained from the patients' medical records.

a. HbA1C level: ___________ %

b. Height: ___________ Centimetre

c. Weight: ___________ Kg

d. LDL: ___________ mg/dL

e. TG: _____________ mg/dL

f. TC: ________________ mg/dL

g. Supplement intake (vitamin B complex): __________ 0 No 1 Yes

h. Supplement intake (vitamin B6): __________ 0 No 1 Yes

i. Starting treatment modality for diabetes
0 Medical nutrition treatment 1 Oral hypoglycemics 2 Insulin

j. Current treatment modality for diabetes:
0 Medical nutrition treatment 1Oral medications 2 Insulin 3. Oral Medications plus insulin 4. Oral plus injectable medications

k. Do you take herbal supplements 0 No 1Yes __________ If yes specify name_______________________ and duration ________Yrs.

l. Have you been diagnosed diabetic complications since DM diagnosis?

1. Nephropathy
2. Retinopathies
3. Diabetic foot/ ulcer
4. Peripheral artery disease
5. Depression
6. Sexual dysfunction

7. None
8. Others as diagnosed by the physician ________

SECTION C: Smoking

13. What is your smoking status?

0 Never smoker 1 Past smoker 2 Current smoker

Note: If you are a never smoker, please skip to section E.

13.1 If you are a current or past smoker.

a. how old were you when have first smoked ever? __________Years
b. how old were you when you started smoking regularly? __________Years

13.2 If you are a past smoker.

how old were you when you stopped smoking ever since? __________Years

14. If you are a current smoker, what type of tobacco do you consume?

0 Tobacco cigarette
1 Kreteks
2 Pipe full of tobacco
3 Shisha (Nargila, argileh, waterpipe / hookah)
4 Cigar
5 E-cigarettes (Vape)
6 Other, please specify: ___________

15. If you are a current smoker, how frequent do you smoke?

0 Less than daily 1 Daily

16. On average, what is your daily consumption of smoking in the past 7 days?

____

17. If you are a current smoker, have you ever tried quitting smoking? If yes, how many times and for how long?

0 No 1 Yes ___________ times, ___________ (days, months, or years)

SECTION D: Physical activity

This section will ask you about the time you spend doing different types of physical activity in a typical week. Please answer these questions even if you do not consider yourself to be a physically active person.

Think first about the time you spend doing work. Think of work as the things that you have to do such as paid or unpaid work, study/training, household chores, harvesting food/crops, fishing or hunting for food, seeking employment. *[Insert other examples if needed].* In answering the following questions 'vigorous-intensity activities' are activities that require hard physical effort and cause large increases in breathing or heart rate, 'moderate-intensity activities' are activities that require moderate physical effort and cause small increases in breathing or heart rate.

18. **Does your work involve vigorous-intensity activity that causes large increases in breathing or heart rate like *[carrying or lifting heavy loads, digging or construction work*] for at least 10 minutes continuously?**
    1 Yes 0 No If no, go to question 21

19. **In a typical week, on how many days do you do vigorous-intensity activities as part of your work?**
    - Number of days: ___________

20. **How much time do you spend doing vigorous-intensity activities at work on a typical day?**
    Minutes: ___________ (If hours convert into minutes)

21. **Does your work involve moderate-intensity activity that causes small increases in breathing or heart rate such as brisk walking *[or carrying light loads*] for at least 10 minutes continuously?**
    1 Yes 0 No If no, go to question 24

22. **In a typical week, on how many days do you do moderate-intensity activities as part of your work?**
    Number of days: ___________

23. **How much time do you spend doing moderate-intensity activities at work on a typical day?**
    Minutes: ___________ (If hours convert into minutes)

The next questions exclude the physical activities at work that you have already mentioned.

Now I would like to ask you about the usual way you travel to and from places. For example, to work, for shopping, to market, to place of worship.

24. **Do you walk or use a bicycle (*pedal cycle*) for at least 10 minutes continuously to get to and from places?**
    1 Yes 0 No If no, go to question 27

25. **In a typical week, on how many days do you walk or bicycle for at least 10 minutes continuously to get to and from places?**
    Number of days: ___________

26. **How much time do you spend walking or bicycling for travel on a typical day?**
    Minutes: ___________ (If hours convert into minutes)

The next questions exclude the work and transport activities that you have already mentioned.
Now I would like to ask you about sports, fitness and recreational activities (leisure).

27. **Do you do any vigorous-intensity sports, fitness or recreational (*leisure*) activities that cause large increases in breathing or heart rate like [*running or football,]* for at least 10 minutes continuously?**
    1 Yes   0 No   If no, go to question 30

28. **In a typical week, on how many days do you do vigorous-intensity sports, fitness or recreational (*leisure*) activities?**
    Number of days: ___________

29. **How much time do you spend doing vigorous-intensity sports, fitness or recreational activities on a typical day?**
    Minutes: ___________ (If hours convert into minutes)

30. **Do you do any moderate-intensity sports, fitness or recreational *(leisure*) activities that causes a small increase in breathing or heart rate such as brisk walking, (*cycling, swimming, volleyball*) for at least 10 minutes continuously?**
    1 Yes   0 No   If no, go to question 33

31. **In a typical week, on how many days do you do moderate-intensity sports, fitness or recreational (*leisure*) activities?**
    Number of days: ___________

32. **How much time do you spend doing moderate-intensity sports, fitness or recreational (*leisure*) activities on a typical day?**
    Minutes: ___________ (If hours convert into minutes)

The following question is about sitting or reclining at work, at home, getting to and from places, or with friends including time spent [sitting at a desk, sitting with friends, travelling in car, bus, train, reading, playing cards, or watching television], but do not include time spent sleeping.

33. **How much time do you usually spend sitting or reclining on a typical day?**
Minutes: ___________ (If hours convert into minutes)
